# What Contributes to the Minimum Inhibitory Concentration? Beyond β-Lactamase Gene Detection in *Klebsiella pneumoniae*

**DOI:** 10.1093/infdis/jiae204

**Published:** 2024-04-24

**Authors:** Alyssa K W Maclean, Stacey Morrow, Fang Niu, Nancy D Hanson

**Affiliations:** Department of Medical Microbiology and Immunology, CRISS 2; Center for Antimicrobial Resistance and Epidemiology; Department of Medical Microbiology and Immunology, CRISS 2; Center for Antimicrobial Resistance and Epidemiology; Department of Clinical Research and Evaluative Sciences, Creighton University School of Medicine, Omaha, Nebraska; Department of Medical Microbiology and Immunology, CRISS 2; Center for Antimicrobial Resistance and Epidemiology

**Keywords:** β-lactamase, *Klebsiella pneumoniae*, OmpK35, OmpK36, PhoE, ESBL, AmpC

## Abstract

**Background:**

*Klebsiella pneumoniae* is capable of resistance to β-lactam antibiotics through expression of β-lactamases (both chromosomal and plasmid-encoded) and downregulation of outer membrane porins. However, the extent to which these mechanisms interplay in a resistant phenotype is not well understood. The purpose of this study was to determine the extent to which β-lactamases and outer membrane porins affected β-lactam resistance.

**Methods:**

Minimum inhibitory concentrations (MICs) to β-lactams and inhibitor combinations were determined by agar dilution or Etest. Outer membrane porin production was evaluated by Western blot of outer membrane fractions. β-lactamase carriage was determined by whole genome sequencing and expression evaluated by real-time reverse-transcription polymerase chain reaction.

**Results:**

Plasmid-encoded β­-lactamases were important for cefotaxime and ceftazidime resistance. Elevated expression of chromosomal SHV was important for ceftolozane-tazobactam resistance. Loss of outer membrane porins was predictive of meropenem resistance. Extended-spectrum β-lactamases and plasmid-encoded AmpCs (pAmpCs) in addition to porin loss were sufficient to confer resistance to the third-generation cephalosporins, piperacillin-tazobactam, ceftolozane-tazobactam, and meropenem. pAmpCs (CMY-2 and DHA) alone conferred resistance to piperacillin-tazobactam.

**Conclusions:**

Detection of a resistance gene by whole genome sequencing was not sufficient to predict resistance to all antibiotics tested. Some β-lactam resistance was dependent on the expression of both plasmid-encoded and chromosomal β-lactamases and loss of porins.

## Teaser

β-lactamases are the major resistance mechanism associated with β-lactam resistance. However, chromosomal β-lactamases and porins are important resistance mechanisms that contribute to the minimum inhibitory concentration (MIC). This article identifies the interplay among chromosomal and plasmid-encoded mechanisms and their contribution to the MIC.

## Graphical Abstract

**Graphical Abstract:**
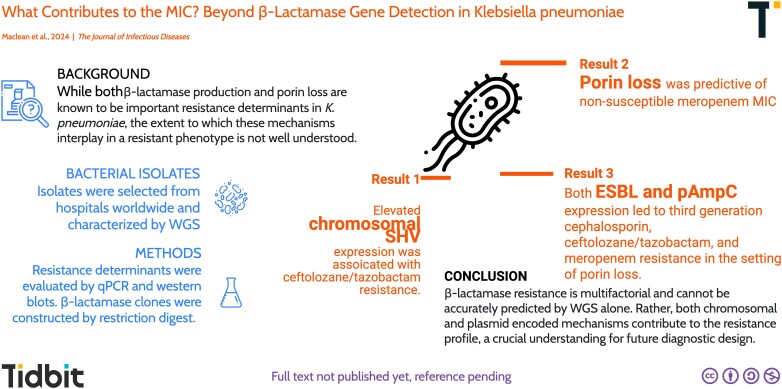

Since the initial Centers for Disease Control and Prevention antimicrobial trends report in 2013, incidence of extended-spectrum β-lactamase (ESBL)–producing Enterobacterales has increased by 50% [1]. *Klebsiella pneumoniae* is particularly concerning as a multidrug-resistant isolate, due to plasmids that can encode ESBLs and/or AmpCs [2]. Current treatment recommendations for these infections include use of carbapenems or β-lactam/β-lactamase inhibitor combinations [3]. However, carbapenem resistance extends beyond isolates that produce a carbapenemase [4]. Noncarbapenemases, like ESBLs and plasmid-encoded AmpCs (pAmpCs), are hypothesized to confer carbapenem resistance when paired with loss of outer membrane porins [5]. β-lactam/β-lactamase inhibitor combinations, too, are less reliable, especially against *K pneumoniae* [6]. When resistance prevents the use of a β-lactam, non-β-lactam antibiotics are recommended [3] but are associated with more toxic side effects, and co-resistance is often found in multidrug-resistant isolates [7].

The contribution of ESBLs to the β-lactam minimum inhibitory concentrations (MICs) observed in *K pneumoniae* has been documented [8, 9]. However, fewer data regarding the impact of pAmpCs on β-lactam MICs are available, despite research indicating their ability to hydrolyze a wide variety of β-lactam antibiotics [10]. Additionally, the contribution of outer membrane porins to the resistance profile of *K pneumoniae* isolates expressing ESBLs or pAmpCs against the newer β-lactam/β-lactamase inhibitor combinations is unknown. Previous work with these newer agents has relied on genomic rather than phenotypic analysis of porin function [11]. The role of porins has been established against carbapenems; however, these studies relied on the use of sodium dodecyl sulphate–polyacrylamide gel electrophoresis to establish porin loss [12–14]. In the absence of specific porin-directed antibodies, the role of each porin cannot be definitively established due to similarity in size and lack of resolution. *Klebsiella pneumoniae* produces 3 outer membrane porins, OmpK35, OmpK36, and PhoE, that are associated with cephalosporin and/or carbapenem resistance in combination with β-lactamases [4, 14].

In addition to plasmid-encoded enzymes and/or porin production, *K pneumoniae* harbors a chromosomally encoded broad-spectrum β-lactamase, SHV-1, conferring intrinsic resistance to penicillins [15]. This β-lactamase may be problematic for ceftolozane-tazobactam as low-level SHV-1 production has been shown to hydrolyze tazobactam [16]. Some *K pneumoniae* strains have been shown to hyperproduce SHV-1, exhibiting an ESBL phenotype [17]. Despite its ubiquity, the role SHV-1 plays on newer β-lactam susceptibility is unknown.

Although it is well accepted that interplay among mechanisms of resistance occurs, the impact on β-lactam susceptibility from 1 or more β-lactamases (plasmid or chromosomally encoded) paired with porin loss has not been clearly defined. Questions addressed during this study include: Do certain combinations of mechanisms influence β-lactam drugs differently? Is resistance limited to a specific class of β-lactamases paired with porin loss? Are all carbapenems or newer β-lactam/β-lactamase inhibitor combinations influenced by the absence of porins?

We hypothesized that the contribution of SHV-1, ESBLs, and/or AmpC production on the susceptibility of clinical isolates of *K pneumoniae* will be both porin and drug dependent and that these combinations impact susceptibility for some drugs but not others.

## METHODS

### Bacterial Strains

Nineteen pAmpC- and ESBL-producing *K pneumoniae* isolates were selected for evaluation based on β-lactamase production and β-lactam MICs. Kp 23 was selected as the comparator due to its susceptible phenotype and lack of plasmid-encoded resistance genes. Clinical isolate KPM 20 was selected for cloning experiments due to its susceptible phenotype and undetectable OmpK35, OmpK36, and PhoE by Western blot. The genomes for Kp 23 and KPM 20 have been published elsewhere [18].

### Antimicrobial Susceptibility Testing

Antibiotic susceptibility tests for the clinical isolates were determined by agar dilution. Susceptibility testing for the *K pneumoniae* clones were determined by Etest. ESBL confirmatory tests for the SHV clones were performed using disk diffusion. All antimicrobial susceptibility tests were performed and interpreted according to Clinical and Laboratory Standards Institute guidelines [19]. Etests were performed following the package insert.

### β-Lactamase Identification

Isolates were initially screened for the β-lactamase genes using the Streck ARM-D *ampC* ID kit and ARM-D β-lactamase ID kit. β-lactamase genes not identified by polymerase chain reaction (PCR) were identified by whole genome sequencing (WGS).

### Whole Genome Sequencing

The method used for WGS is described in Supplementary Data.

### RNA Analysis by Real-Time Reverse-Transcription PCR

Evaluation of RNA expression has been previously described [20–22] (Supplementary Data). Primer sequences are available in Supplementary Table 1.

### Western Blot

Protein isolation and Western blotting was performed as described by Suelter et al [23]. (Supplementary Table 2, Supplementary Figure 1) [24].

### Cloning Strategy

All β-lactamase genes were cloned containing both the structural gene and promoter (Supplementary Table 3) [25–27] and transformed into Kp 23 and KPM 20 (Supplementary Figure 2) as described in Supplementary Data [28, 29].

## RESULTS

### Does β-Lactamase Gene Detection Predict Susceptibility?

We compared the presence of the β-lactamase gene with the MICs for ceftriaxone, cefotaxime, ceftazidime, cefepime, piperacillin-tazobactam, ceftolozane-tazobactam, ertapenem, and meropenem in 20 different clinical isolates of *K pneumoniae*. The presence of a plasmid-encoded β-lactamase was significantly associated with a resistant cefotaxime and ceftazidime MIC (*P* < .001 and *P* < .005, respectively), regardless of whether that β-lactamase was a pAmpC or ESBL. All 19 isolates expressing an ESBL were resistant to ceftriaxone. However, no correlation with β-lactamase presence and MIC was noted for cefepime, ceftolozane-tazobactam, piperacillin-tazobactam, ertapenem, or meropenem, suggesting that an alternate β-lactamase, variability in the expression, or another resistance mechanism may be involved (Table 1).

**Table 1.** β-Lactam Minimum Inhibitory Concentrations of *Klebsiella pneumoniae* Clinical Isolates

Isolate	β-Lactamase	MIC, µg/mL (S/I/R)^a^							
		CRO	CTX	CAZ	FEP	C/T	P/T	MEM	ERT
Kp 23	SHV-11	≤0.06 (S)	0.032 (S)	0.064 (S)	0.047 (S)	0.64 (S)	1.5 (S)	0.047 (S)	≤0.06 (S)
KPM 1	None	0.5 (S)	≤1 (S)	1.5 (S)	6 (SDD)	0.5 (S)	≥128 (R)	2 (I)	16 (R)
KPM 5	CTX-M-14 SHV-11 TEM-1 CMY-2-like	16 (R)	≥64 (R)	0.25 (S)	1.5 (S)	0.25 (S)	≤4 (S)	0.06 (S)	≤0.06 (S)
KPM 8	CTX-M-15 **SHV-27**^b^ TEM-1	128 (R)	≥32 (R)	64 (R)	≥512 (R)	0.25 (S)	2 (S)	0.12 (S)	≤0.06 (S)
KPM 9	CTX-M-15 **SHV-27** TEM-1	128 (R)	≥32 (R)	64 (R)	≥512 (R)	0.25 (S)	2 (S)	0.12 (S)	0.12 (S)
KPM 10	CTX-M-15 OXA-1 **SHV-28** TEM-1	64 (R)	≥32 (R)	8 (R)	4 (SDD)	0.25 (S)	2 (S)	0.06 (S)	≤0.06 (S)
KPM 17	CTX-M-15 OXA-1 SHV-11 TEM-1	64 (R)	≥64 (R)	12 (I)	6 (SDD)	1 (S)	32 (I)	0.12 (S)	≤0.06 (S)
KPM 18	CTX-M-15 **SHV-28** OXA-1 TEM-1	≥128 (R)	≥64 (R)	24 (R)	32 (R)	1 (S)	32 (I)	0.06 (S)	≤0.06 (S)
KPM 20	SHV-11	0.5 (S)	1 (S)	1.5 (S)	1.5 (S)	1 (S)	128 (R)	0.5 (S)	2 (R)
KPM 21	DHA^c^ SHV-60	0.5 (S)	64 (R)	64 (R)	0.25 (S)	2 (S)	8 (S)	2 (I)	0.25 (S)
KPM 23	CMY-2 **SHV-187** TEM-1	16 (R)	12 (R)	24 (R)	0.38 (S)	4 (I)	32 (I)	0.06 (S)	0.12 (S)
KPM 26	CTX-M-19 SHV-1 TEM-1	32 (R)	256 (R)	32 (R)	4 (SDD)	4 (I)	8 (S)	0.06 (S)	0.12 (S)
KPM 29	CMY-31 **SHV-5**	64 (R)	128 (R)	512 (R)	2 (S)	8 (R)	512 (R)	0.06 (S)	0.12 (S)
KPM 30	CTX-M-15 OXA-1 TEM-1 SHV-1	≥128 (R)	≥64 (R)	≥128 (R)	≥32 (R)	8 (R)	≥128 (R)	4 (R)	32 (R)
KPM 32	CTX-M-15 OXA-1 **SHV-12**	≥128 (R)	≥64 (R)	≥128 (R)	≥32 (R)	8 (R)	≥128 (R)	4 (R)	16 (R)
KPM 42	DHA-1 **SHV-12** TEM-1	32 (R)	128 (R)	≥512 (R)	128 (R)	64 (R)	≥512 (R)	0.47 (S)	0.5 (S)
KPM 43	CTX-M-14 DHA-1 SHV-11 TEM-1	≥128 (R)	≥64 (R)	128 (R)	≥32 (R)	64 (R)	≥128 (R)	4 (R)	≥32 (R)
KPM 44	CTX-M-14 DHA-1 SHV-11 TEM-1	≥128 (R)	≥512 (R)	64 (R)	≥512 (R)	64 (R)	≥512 (R)	16 (R)	≥32 (R)
KPM 60	CTX-M-15 OXA-1 SHV-11 TEM-1	≥128 (R)	≥64 (R)	≥128 (R)	≥32 (R)	≥512 (R)	≥128 (R)	8 (R)	32 (R)
KPM 61	CTX-M-15 OXA-1 SHV-1 TEM-1	≥128 (R)	≥32 (R)	≥256 (R)	≥256 (R)	≥256 (R)	96 (I)	3 (I)	32 (R)

Abbreviations: CAZ, ceftazidime; CRO, ceftriaxone; C/T, ceftolozane-tazobactam; CTX, cefotaxime; ERT, ertapenem; FEP, cefepime; MEM, meropenem; P/T, piperacillin-tazobactam; SDD, succeptible dose-dependent.

^a^Susceptible/intermediate/resistant/succeptible dose-dependent (SDD) as determined by Clinical and Laboratory Standards Institute breakpoints and agar dilution MIC.

^b^SHV extended-spectrum β-lactamases are indicated in bold.

^c^DHA allele does not align with known amino acid sequences and likely represents a previously unidentified DHA.

### WGS-Identified Plasmid-Encoded ESBLs and AmpCs

Whole genome sequencing was performed to identify additional β-lactamases not detected by PCR, identify specific SHV genes, and identify amino acid changes in the porin genes of the clinical isolates compared to Kp23. All AmpC and ESBL genes identified by PCR were confirmed with WGS, except for 1 isolate, KPM 5, which had a *bla_CMY-2_* by PCR but not by WGS (Supplementary Table 4). Of these 20 strains, 17 isolates had an ESBL or pAmpC. Of these, 4 carried *bla_CTX-M-14-like_*, 9 carried *bla_CTX-M-15-like_*, 4 carried *bla_DHA-1-like_*, and 3 carried *bla_CMY-2-like_*. Two isolates had both *bla_DHA-1_* and *bla_CTX-M-14_*, and 1 had both *bla_CMY-2-like_* and *bla_CTX-M-14_*.

Specific SHV alleles were determined for each isolate. Eleven isolates had broad-spectrum SHV alleles (7 *bla_SHV-11_*, 3 *bla_SHV-1_*, and 1 *bla_SHV-60_*) and 7 had SHV ESBLs (Table 1). One isolate had no detectable SHV sequence but had detectable *bla_SHV-72_* and *bla_OXA-2_*. None of the SHV genes detected were inhibitor resistant [30]. Thirteen isolates carried *bla_TEM-1_* and 7 had *bla_OXA-1_*.

### Factors Contributing to MIC in Clinical Isolates

**Figure 1.**
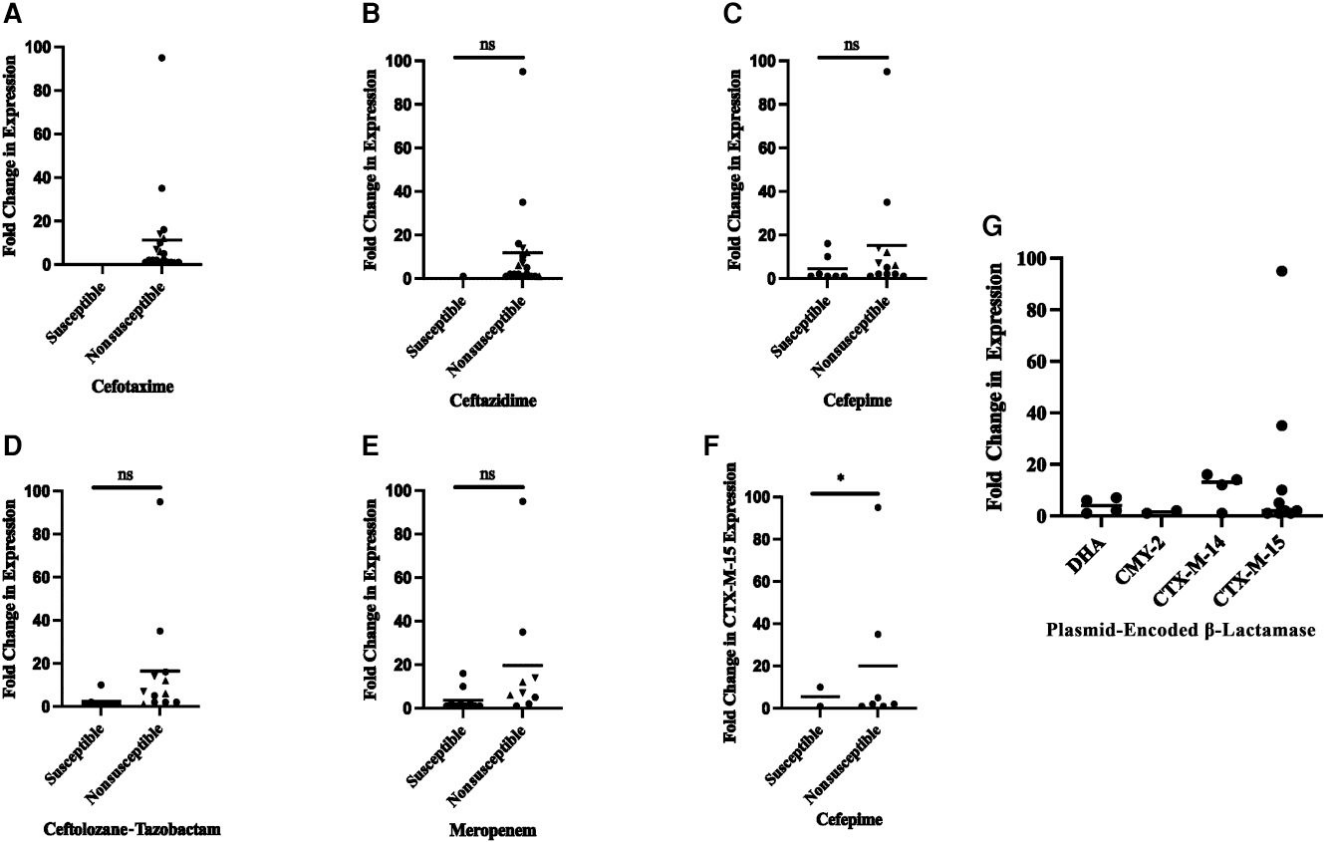
Fold change in extended-spectrum β-lactamase and AmpC expression in clinical isolates. Fold change in RNA expression of all plasmid-encoded β-lactamases of isolates that are susceptible or nonsusceptible to cefotaxime (*A*), ceftazidime (*B*), cefepime (*C*), ceftolozane-tazobactam (*D*), and meropenem (*E*). *F*, *bla_CTX-M-15_* expression in cefepime-susceptible and -nonsusceptible isolates. *G*, Expression of β-lactamases by enzyme family. Significance determined by Wilcoxon 2-sample test. *P < .5. Mean expression is noted by the cross-bar. Abbreviation: ns, not significant.

Fold change in β-lactamase expression was assessed for all isolates to examine the effect of β-lactamase expression level on β-lactam MIC. When all enzymes were grouped together, there was a trend toward increased expression in nonsusceptible isolates, but these data were not statistically significant (Figure 1). When the fold change in expression for each individual enzyme was compared for each antibiotic, there was a statistically significant increase in the fold *bla_CTX-M-15_* expression among cefepime-nonsusceptible isolates (Figure 1*F*). OXA-1 production did not seem to have an impact on cefepime MICs as isolate KPM 61, which had a cefepime MIC of ≥256 µg/mL, had very little detectable OXA-1 (Supplementary Figure 3, Table 1). Higher OXA-1 production was noted in some isolates with higher cefepime MICs. Those isolates, in addition to CTX-M-15, also co-produced other ESBLs such as SHV-28 (KPM 18) or SHV-12 (KPM 32). The role of porins is also implicated in some of these isolates (Table 2) as 4 of 5 resistant isolates with OXA-1 were porin deficient. No other associations between plasmid-encoded β-lactamase expression and β-lactam MIC were noted.

**Table 2.** Mutations in OmpK35, OmpK36, PhoE, and TolC

Isolate	Outer Membrane Porin Phenotype	OmpK35	OmpK36	PhoE
Kp 23	Present	Comparator	Comparator	Comparator
KPM 1	Absent	No mutations	D198X	No mutations
KPM 5	Present	No mutations	T183A, ins184L, G191T, F200Y, H220N, N224L, ins229S, R229K, D231A, K232L, delF267_E272, S273A, D274G, I276L, delS277, I312L, L320I, E349D, D351S, R354H, R355N	No mutations
KPM 8	Present	No mutations	T183A, ins184L, G191T, F200Y, H220N, N224L, ins229S, R229K, D231A, K232L, delF267_E272, S273A, D274G, I276L, delS277, I312L, L320I, E349D, D351S, R354H, R355N	No mutations
KPM 9	Present	No mutations	T183A, ins184L, G191T, F200Y, H220N, N224L, ins229S, R229K, D231A, K232L, delF267_E272, S273A, D274G, I276L, delS277, I312L, L320I, E349D, D351S, R354H, R355N	No mutations
KPM 10	Present	No mutations	T183A, ins184L, G191T, F200Y, H220N, N224L, ins229S, R229K, D231A, K232L, delF267_E272, S273A, D274G, I276L, delS277, I312L, L320I, E349D, D351S, R354H, R355N	No mutations
KPM 17	Present	No mutations	T183A, ins184L, G191T, F200Y, H220N, N224L, ins229S, R229 K, D231A, K232L, delF267_E272, S273A, D274G, I276L, delS277, I312L, L320I, E349D, D351S, R355N	No mutations
KPM 18	Present	V356X	T183A, ins184L, G191T, F200Y, H220N, N224L, ins229S, R229K, D231A, K232L, delF267_E272, S273A, D274G, I276L, delS277, I312L, L320I, E349D, D351S, R354H, R355N	No mutations
KPM 20	Absent	No mutations	F170X	No mutations
KPM 21	Present	No mutations	delT183_S184, P185A, A192W, L193S, Y209W, N224T, G225D, D226E, R229S, L230V, D231P, K232A, ins232L, T256S, delF267_E272, S273A, D274G, I276L, delS277, I312L, ins313R, L320I, E349D, D351S, R355N	No mutations
KPM 23	Present	No mutations	T86V, S88G, S89T, S90D, D91K, A93S, delG182_T183, S184D, P185M, L193Q, L204V, Y209W, N224T, G225D, R229N, D231V, K232L, N237D, T256S, del267F_272E, S273A, D274G, I276L, delS277, I312L, ins313R, L320I, E349D, D351S, R355N	
KPM 26	Present	No mutations	delT183_S184, P185A, A192W, L193S, Y209W, N224T, G225D, D226E, R229S, L230V, D231P, K232A, ins232L, T256S, delF267_E272, S273A, D274G, I276L, delS277, I312L, ins313R, L320I, R355K	No mutations
KPM 29	Present	No mutations	No mutations	N28S
KPM 30	Absent	D54N	Start codon mutation	No mutations
KPM 32	Absent	N87X	Could not be determined due to contig break across gene	No mutations
KPM 42	Present	Start codon mutation	T183A, ins184L, G191T, F200Y, H220N, N224L, ins229S, R229K, D231A, K232L, delF267_E272, S273A, D274G, I276L, delS277, I312L, L320I, E349D, D351S, R354H, R355N	No mutations
KPM 43	Absent	S253X	L102X	No mutations
KPM 44	Absent	S233X	L102X	No mutations
KPM 60	Absent	No mutations	Start codon mutation	No mutations
KPM 61	Absent	E132K	Q66X	No mutations

**Figure 2.**
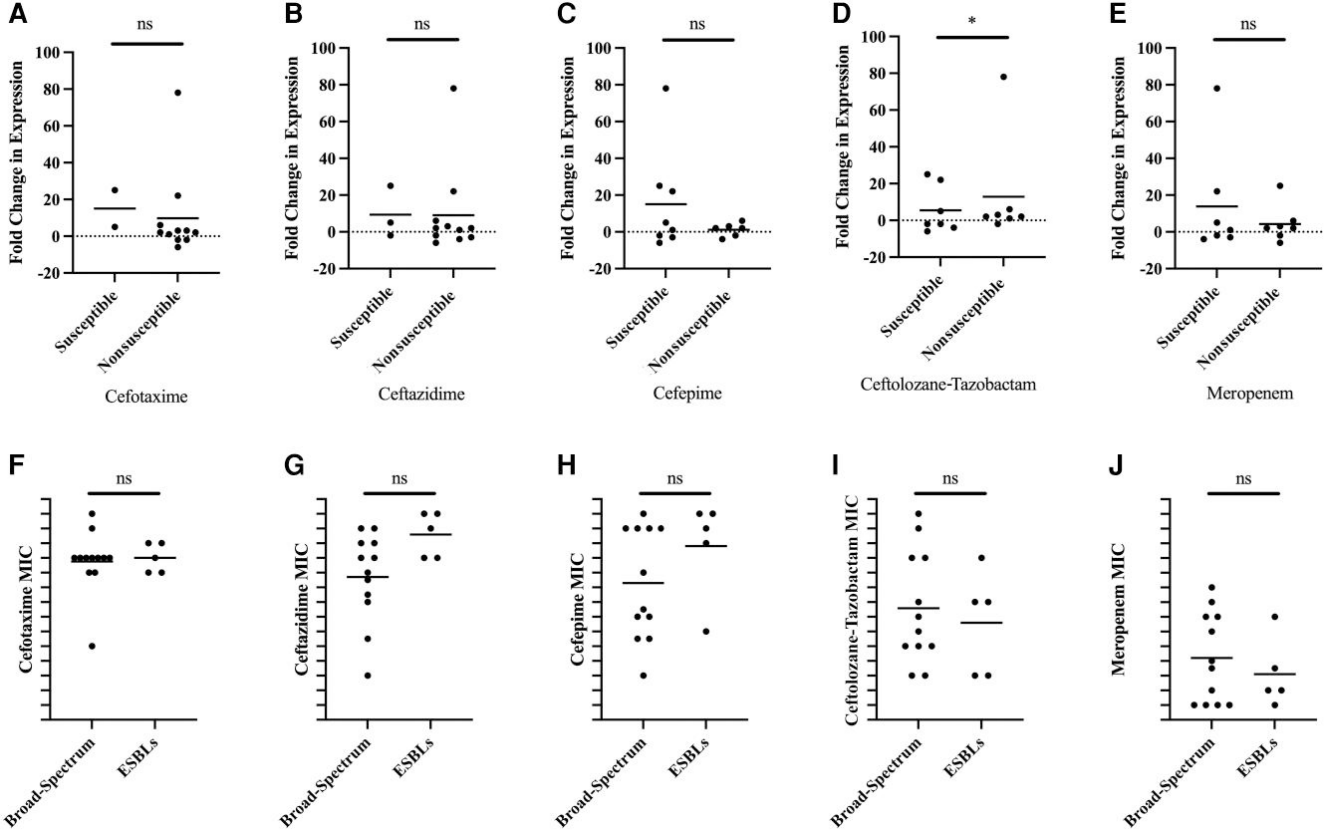
Fold change in SHV expression in clinical isolates. Fold change in SHV RNA expression was compared between susceptible and nonsusceptible isolates for cefotaxime (*A*), ceftazidime (*B*), cefepime (*C*), ceftolozane-tazobactam (*D*), and meropenem (*E*). Cross-bar represents mean RNA expression. Minimum inhibitory concentration was charted for both broad-spectrum SHVs and extended-spectrum β-lactamases for cefotaxime (*F*), ceftazidime (*G*), cefepime (*H*), ceftolozane-tazobactam (*I*), and meropenem (*J*). Significance was determined by Wilcoxon 2-sample test. *P < .05. Abbreviations: ESBL, extended-spectrum β-lactamase; MIC, minimum inhibitory concentration; ns, not significant.

The isolates evaluated in this study produced both broad-spectrum and extended-spectrum SHVs, but each isolate only encoded 1 SHV by WGS. Regardless of whether the SHV gene was a broad or extended-spectrum SHV, there was a statistically significant increase in *bla_SHV_* expression among isolates that were nonsusceptible to ceftolozane-tazobactam (Figure 2*D*). There was no difference between the ESBLs and broad-spectrum SHV β-lactamases.

**Figure 3.**
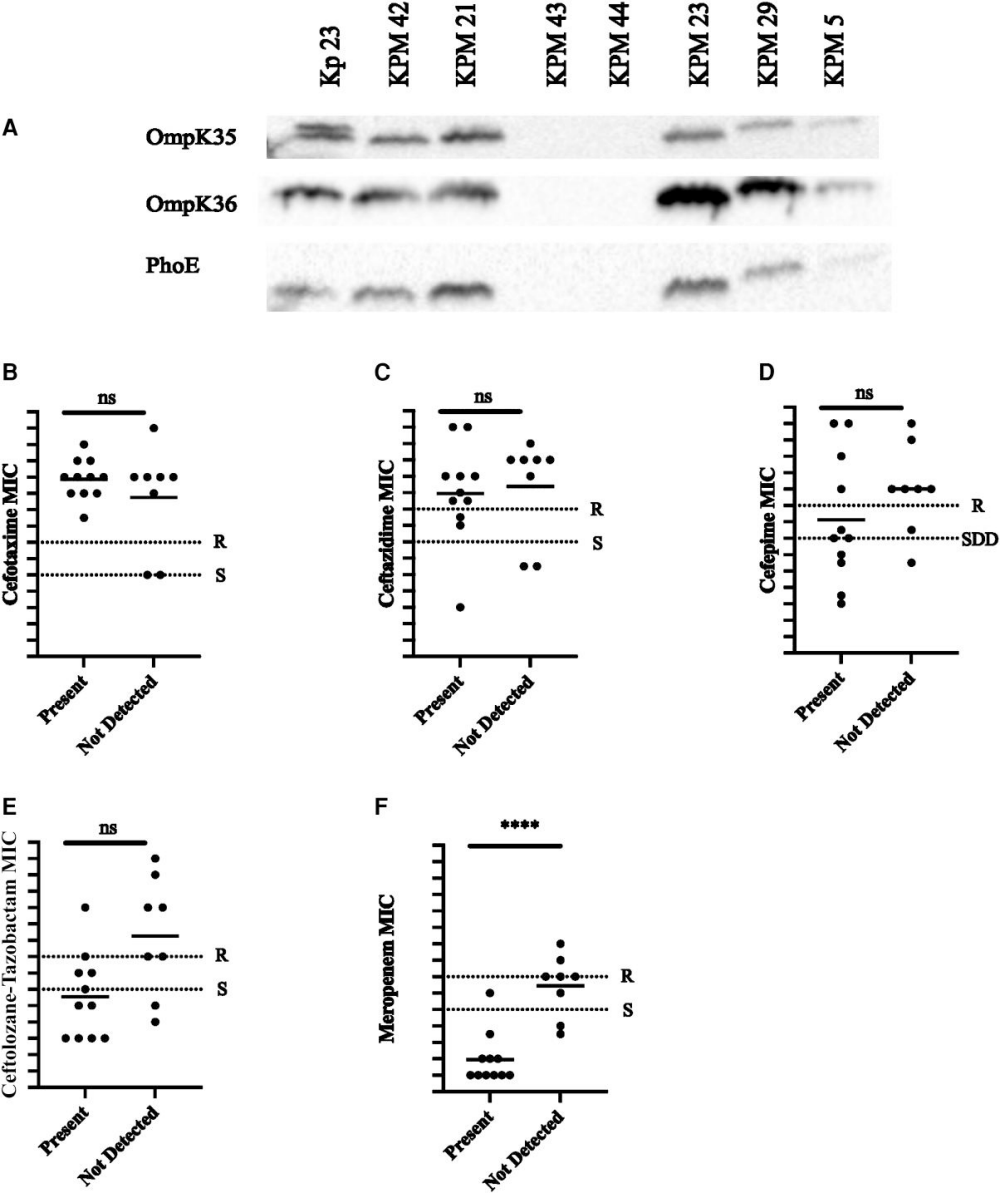
Difference in β-lactam minimum inhibitory concentrations (MICs) in porin-deficient strains. *A*, Representative blot of OmpK35, OmpK35, and PhoE in outer membrane fractions of *Klebsiella pneumoniae.* MIC (log_2_ transformed) for cefotaxime (*B*), ceftazidime (*C*), cefepime (*D*), ceftolozane-tazobactam (*E*), and meropenem (*F*) was determined in isolates that had OmpK35, OmpK36, and PhoE in the outer membrane (grouped as “Present”) and in isolates lacking OmpK35, OmpK36, and PhoE in the outer membrane (grouped as “not detected”). Cross-bars represent average MIC in each group. Dotted lines indicate Clinical and Laboratory Standards Institute breakpoints for resistance and susceptibility for each antibiotic. Significance determined by Fisher exact test. ****P = .0008. Abbreviations: MIC, minimum inhibitory concentration; ns, not significant; R, resistant; S, susceptible; SDD, susceptible dose-dependent.

The presence of the porins in the outer membrane was evaluated by isolation of outer membrane fractions and Western blot analysis for OmpK35, OmpK36, and PhoE. Isolates were grouped by presence or absence of all 3 porins and the MIC to each antibiotic was compared (Figure 3). For ceftazidime, cefepime, and ceftolozane-tazobactam, there was a trend toward higher MICs in the isolates lacking all 3 porins. There was a statistically significant increase in meropenem MICs among isolates lacking the 3 porins (Figure 3*F*). No association was noted for cefotaxime. Using logistic regression analysis, loss of the outer membrane porins predicted meropenem nonsusceptibility (*P* = .0037). When the presence of a plasmid-encoded enzyme was corrected for, the association between loss of outer membrane porins and meropenem resistance improved (*P* < .0001). All the isolates missing porins were resistant to ertapenem.

The amino acid sequences of the porins were assessed using WGS data and compared to the sequence of Kp 23 (Table 2) [18]. For OmpK35, differences compared to Kp 23 in the porin amino acid sequence were not always present in isolates that were lacking the porin in the outer membrane. One isolate, KPM 42, had detectable OmpK35 in the outer membrane, but by WGS, the first 82 amino acids were replaced with sequences that most closely resembled phage DNA, suggesting a nonfunctional product. For OmpK36, there was a large amount of amino acid sequence variability between the clinical isolates, with 18 of 20 strains encoding differences in the protein, compared to Kp 23. In those isolates lacking OmpK36 in the outer membrane, start codon differences, or nonsense codons were present. Interestingly, 1 strain, KPM 18, had a truncation resulting in a 356-aa product that was present in the outer membrane fraction, suggesting that the shortened length of the protein was still long enough to allow translocation. When OmpK36 was present in the outer membrane, common areas of variability were noted in 10 of 12 strains between amino acids 183 and 355. Only 1 strain had a single amino acid substitution in the PhoE gene that was not correlated with a loss of the porin in the outer membrane. The absence of PhoE in the membrane of some of the isolates was not due to differences observed in in the steady state RNA expression or the amino acid sequence between those isolates and isolates in which the porin was present.

### Interplay Between β-Lactamase Production and Porin Loss

**Figure 4.**
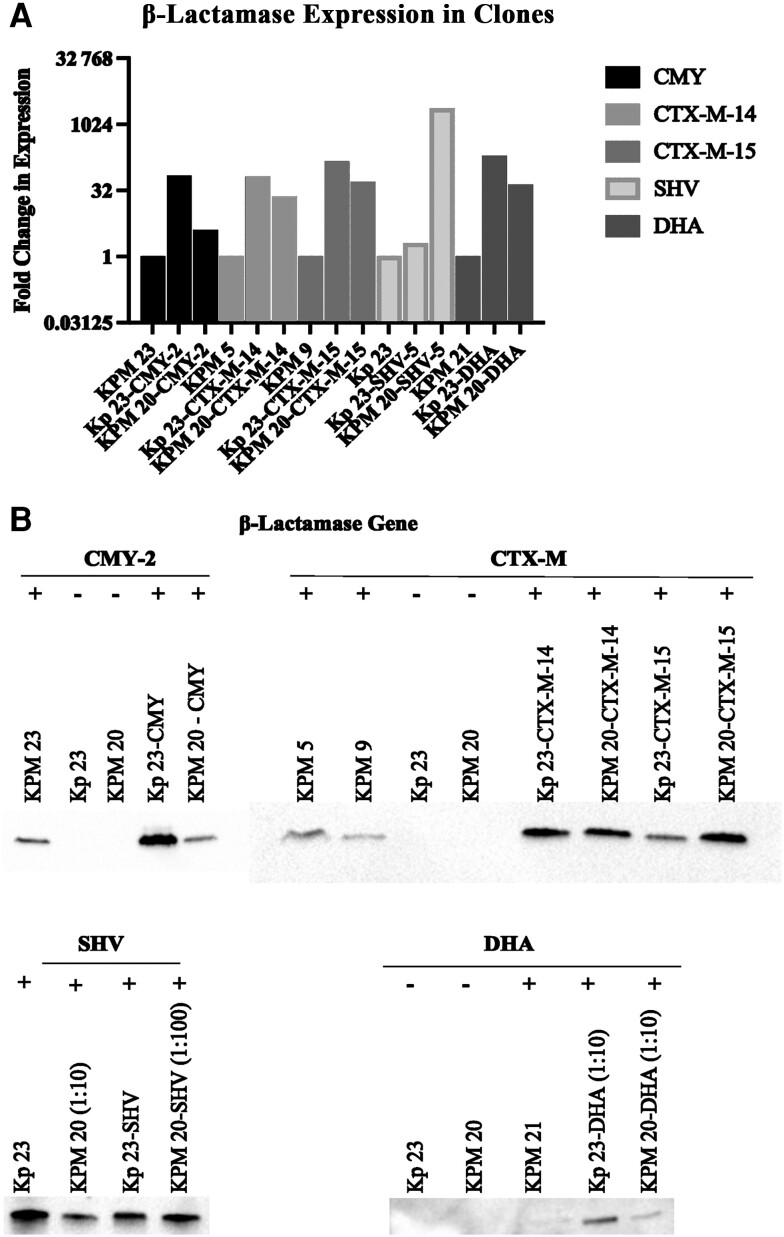
RNA expression and protein production of β-lactamases in β-lactamase clones. β-lactamase expression was confirmed in β-lactamase clones by real-time reverse-transcription polymerase chain reaction (*A*) and by Western blot (*B*) as compared to clinical isolates that are known to produce the β-lactamase but are susceptible to ceftolozane-tazobactam.

To determine the contribution of different β-lactamase expression to β-lactam MIC in the presence and absence of the porins, β-lactamase genes with their native promoters were cloned into Kp 23 (outer membrane porins present) and KPM 20 (no detectable OmpK35, OmpK36, or PhoE) and MICs were determined (Table 3). Variation in the expression and production of the CMY-2 and SHV-5 β-lactamases was observed between the transformants with detectable (Kp 23) and undetectable (KPM 20) porins (Figure 4).

**Table 3.** β-Lactam Minimum Inhibitory Concentrations of *Klebsiella pneumoniae* Porin Clones

Clone	MIC, µg/mL (S/I/R)^a^										
	CRO	CTX	CAZ	FEP	C/T	P/T	CZA	MEM	ERT	I-R	MVB
Kp 23 (parent)	0.047 (S)	0.032 (S)	0.064 (S)	0.047 (S)	0.064 (S)	1.5 (S)	0.064 (S)	0.047 (S)	0.006 (S)	0.125 (S)	0.016 (S)
Kp 23-CMY-2	≥256 (R)	≥32 (R)	≥256 (R)	1.5 (S)	16 (R)	≥256 (R)	0.5 (S)	0.125 (S)	0.38 (S)	0.125 (S)	0.016 (S)
Kp 23-CTX-M-14	≥256 (R)	≥32 (R)	1.5 (S)	12 (SDD)	0.19 (S)	4 (S)	0.064 (S)	0.064 (S)	0.094 (S)	0.125 (S)	0.016 (S)
Kp 23-CTX-M-15	≥256 (R)	≥32 (R)	12 (I)	24 (R)	0.25 (S)	3 (S)	0.125 (S)	0.064 (S)	0.032 (S)	0.125 (S)	0.016 (S)
Kp 23-SHV-5	0.38 (S)	0.5 (S)	0.75 (S)	0.38 (S)	0.5 (S)	16 (S)	0.5 (S)	0.032 (S)	0.032 (S)	0.094 (S)	0.016 (S)
Kp 23-DHA-AmpR	3 (I)	≥32 (R)	≥256 (R)	1.5 (S)	3 (I)	≥256 (R)	0.125 (S)	0.125 (S)	0.032 (S)	0.125 (S)	0.094 (S)
KPM 20 (porin-deficient parent)	1 (S)	1 (S)	1.5 (S)	1.5 (S)	1 (S)	17 (I)	0.125 (S)	0.5 (S)	0.75 (S)	0.125 (S)	0.19 (S)
KPM 20-CMY-2	≥256 (R)	≥32 (R)	≥256 (R)	4 (SDD)	16 (R)	≥256 (R)	1 (S)	2 (I)	≥32 (R)	0.25 (S)	0.5 (S)
KPM 20-CTX-M-14	≥256 (R)	≥32 (R)	24 (R)	≥256 (R)	128 (R)	≥256 (R)	1 (S)	4 (R)	≥32 (R)	0.19 (S)	0.75 (S)
KPM 20-CTX-M-15	≥256 (R)	≥32 (R)	≥256 (R)	≥256 (R)	≥256 (R)	≥256 (R)	1 (S)	2 (I)	≥32 (R)	0.19 (S)	0.38 (S)
KPM 20-SHV-5	≥256 (R)	≥32 (R)	≥256 (R)	≥256 (R)	≥256 (R)	≥256 (R)	1.5 (S)	2 (I)	≥32 (R)	0.38 (S)	1.5 (S)
KPM 20-DHA-AmpR	≥256 (R)	≥32 (R)	≥256 (R)	3 (SDD)	16 (R)	≥256 (R)	0.094 (S)	1.5 (I)	≥32 (R)	0.25 (S)	0.38 (S)

Abbreviations: CAZ, ceftazidime; CRO, ceftriaxone; C/T, ceftolozane-tazobactam; CTX, cefotaxime; CZA, ceftazidime-avibactam; ERT, ertapenem; FEP, cefepime; I-R, imipenem-relebactam; MEM, meropenem; MIC, minimum inhibitory concentration; MVB, meropenem/vaborbactam; P/T, piperacillin-tazobactam.

^a^Susceptible/intermediate/resistant/susceptible dose-dependent as determined by Clinical and Laboratory Standards Institute breakpoints and Etest.

In Kp 23, CMY-2 production led to a resistant phenotype to ceftriaxone, cefotaxime, ceftazidime, piperacillin-tazobactam, and ceftolozane-tazobactam (Table 3). In the porin mutant, KPM 20, resistant MICs were noted for these antibiotics and ertapenem, as well as an intermediate MIC to meropenem and a susceptible dose-dependent MIC for cefepime. When DHA-AmpR was transformed into Kp 23, nonsusceptibility was seen to ceftriaxone, cefotaxime, ceftazidime, piperacillin-tazobactam, and ceftolozane-tazobactam, similarly to CMY-2. In the porin mutant KPM 20, nonsusceptibility was seen to ceftriaxone, cefotaxime, ceftazidime, piperacillin-tazobactam, ceftolozane-tazobactam, ertapenem, and meropenem, and a susceptible dose-dependent phenotype was observed for cefepime. Note that the loss of porins in KPM 20 in the presence of CMY-2 did not change the ceftolozane-tazobactam MIC.

Transformation of CTX-M-14 into Kp 23 led to resistant MICs for ceftriaxone, cefotaxime, and a susceptible dose-dependent MIC for cefepime. When CTX-M-15 was produced in Kp 23, resistant MICs to ceftriaxone, cefotaxime, and cefepime were noted, as well as an intermediate MIC to ceftazidime. In KPM 20 with CTX-M-14 or CTX-M-15, resistance to ceftriaxone, cefotaxime, ceftazidime, cefepime, piperacillin-tazobactam, ceftolozane-tazobactam, and ertapenem was observed, along with a resistant (CTX-M-14) or intermediate (CTX-M-15) MIC to meropenem. Surprisingly, production of an SHV ESBL, SHV-5, in the Kp 23 background did not display an ESBL phenotype like CTX-M-14 or CTX-M-15. Instead, no resistant MICs were noted for any of the antibiotics tested. However, in the porin-deficient parent, SHV-5 displayed an ESBL phenotype with nonsusceptibility to cefotaxime, ceftazidime, cefepime, piperacillin-tazobactam, ceftolozane-tazobactam, ertapenem, and meropenem.

## DISCUSSION

The results of this work highlight the complexity of β-lactam resistance in gram-negative bacteria. To use existing or newly available antibiotics appropriately, we need to be aware that the susceptibility profile presented by the organism is based on the combination of resistant mechanisms. It is clear from this study that gene identification alone cannot determine the impact on the susceptibility profile of an organism.

As has been reported, the clinical isolates and clones that produced ESBLs were resistant to cefotaxime [8], but the contribution of ESBLs to ceftazidime MICs was variable between the clinical isolates and the clones (Table 2, Figure 1). In the porin sufficient clone, ceftazidime resistance only occurred in the CTX-M-15–producing isolate. Carriage of a pAmpC or CTX-M ESBL was sufficient for cefotaxime resistance, regardless of porin status, while resistance to ceftazidime occurred in clones when outer membrane porins were lost. Although SHV-5 is reported in the literature to be an ESBL [31], the SHV-5 clone did not display an ESBL phenotype in *K pneumoniae* unless outer membrane porins were lost, possibly due to the discrepancy in SHV-5 production between the Kp 23 and KPM 20 clones. Interestingly, SHV-5 was the only enzyme that showed large variation in expression between the Kp 23 and KPM 20 backgrounds.

DHA-1 production in porin-deficient clinical isolates of *K pneumoniae* has previously been shown to lead to ceftazidime and cefotaxime resistance [32]. In this study, however, resistance occurred regardless of porin status (Table 3). Ceftazidime and cefotaxime resistance has been previously noted in CMY-2–producing *Escherichia coli* [33, 34], although it was unclear from that study if resistance was due to overproduction of the enzyme or co-occurrence of another resistance mechanism. The data presented in this study indicate that pAmpC β-lactamases CMY-2 (and CMY-2-like) and DHA are sufficient to confer ceftazidime and cefotaxime nonsusceptibility in *K pneumoniae*.

There was a significant increase in *bla_CTX-M-15_* expression among cefepime-nonsusceptible clinical isolates, suggesting that CTX-M-15 contributes to cefepime resistance. Although clinical isolates often carry *bla*_OXA-1_ in addition to CTX-M-15, the Kp 23 clone producing CTX-M-15 alone had a cefepime MIC above the resistant breakpoint. These data and the variability in the production of OXA-1 in the clinical isolates evaluated suggest that OXA-1 contributes little to the cefepime MICs when CTX-M-15 is produced. Prior analysis of purified enzyme demonstrated that CTX-M-15 was capable of hydrolyzing cefepime [35] and conferred resistance when expressed in an *E coli* laboratory strain [36]. The results of this work support the finding of CTX-M-15–mediated cefepime resistance. This observation is crucial in guiding treatment for CTX-M-15–producing organisms, as use of cefepime for these infections is controversial [37].

Against ceftolozane-tazobactam, factors important for resistant MICs included pAmpC production regardless of porin status, ESBL production in the setting of porin loss, and elevated expression of the chromosomal SHV. Against piperacillin-tazobactam, resistance patterns were variable in the clinical isolates with no one mechanism clearly contributing to the MIC. However, piperacillin-tazobactam MICs for the clones deficient in porin production but producing an ESBL resulted in a resistant phenotype. To date, few studies have directly examined the contribution of CMY-2 or DHA to ceftolozane-tazobactam resistance. A survey of British isolates revealed 51% susceptibility to ceftolozane-tazobactam among *K pneumoniae* with a PCR-identified pAmpC β-lactamase, 24 of 26 of which were DHA [38]. A more recent survey of ceftolozane-tazobactam resistance in the United States focused largely on ESBL-producing isolates, but did note ceftolozane-tazobactam resistance in a DHA-producing *Enterobacter* sp [11]. Nonsusceptibility to ceftolozane-tazobactam in these species has concerning implications for use of the β-lactam/β-lactamase inhibitor combination in treating pAmpC-producing organisms.

Survey of ceftolozane-tazobactam resistance in the United States has repeatedly demonstrated the involvement of ESBLs in ceftolozane-tazobactam resistance, with no difference in the porin protein sequences among susceptible and nonsusceptible isolates [11]. The data presented in this study indicate that piperacillin-tazobactam and ceftolozane-tazobactam resistance only occurs in ESBL-producing isolates when outer membrane porins are lost. Studies using isogenic clones have supported the combination of CTX-M-15 and decreased permeability (porin loss and efflux) associated with ceftolozane-tazobactam resistance [39]. While phenotypic testing would identify a susceptible or resistant phenotype, molecular screening alone could not correctly identify resistant isolates as testing for porin loss in clinical laboratories is unavailable [19]. The other β-lactam/β-lactamase inhibitor combinations tested remained active against these isolates, suggesting that these antibiotics would be a better choice in treating ESBL-producing *K pneumoniae*.

SHV β-lactamases are encoded on the chromosome of virtually all *K pneumoniae* [15]. While this enzyme is known to confer resistance to the penicillins and aminopenicillins [40], the contribution of broad-spectrum chromosomal SHVs to other β-lactam MICs is not well established. In this analysis, there was a significant increase in RNA expression of *bla_SHV_* among isolates that were nonsusceptible to ceftolozane-tazobactam, regardless of whether the SHV was broad spectrum or an ESBL. A recent study showed increased piperacillin-tazobactam MICs due to high-level production of SHV [41]. The high levels of SHV most likely impact the ability of tazobactam to inhibit the SHV enzyme. Each of the nonsusceptible isolates also produced at least 1 additional plasmid-encoded β-lactamase. In these isolates, trapping of tazobactam by SHV would leave ceftolozane vulnerable to hydrolysis by the other enzyme. A similar mechanism has been proposed for meropenem resistance in an *E coli* strain that produced both CMY-2 and CTX-M-15 [42]. The contribution of SHV could help explain the discrepancy in ceftolozane-tazobactam susceptibility between *K pneumoniae* and *E coli* [6].

In both the clinical isolates and clones, porin loss was predictive of resistant MICs to meropenem. Previous work has implicated OmpK36, PhoE, and/or OmpK35 loss in β-lactam resistance when paired with an ESBL [12] or pAmpC [13]. Prior research regarding the role of porin loss in *K pneumoniae* meropenem resistance is conflicting. Some studies have revealed increased meropenem MICs in mice infected with knockout or mutant strains of OmpK36 [43, 44]. However, those studies used isolates that produced KPC-2, so the effect of OmpK36 is confounded by the presence of the carbapenemase. Conversely, other studies of clinical isolates that produce ESBLs in the setting of porin loss demonstrated susceptibility to meropenem [45, 46]. As with other studies, strains that were deficient in outer membrane porins were also resistant to ertapenem [46]. The data in the current study clearly support that porin loss contributes to meropenem resistance in *K pneumoniae* when paired with an ESBL or pAmpC.

Truncations and/or insertion sequences can interrupt a porin coding sequence and likely correlate with lack of function [46, 47]. However, given the findings in this study, use of WGS to predict porin loss may not be reliable. While nonsense or start codon mutations were associated with loss of OmpK35 5/8 strains and OmpK36 in 7 of 8 strains, amino acid variations were not present in all strains lacking outer membrane porins. In 10 strains, variability was seen in the *ompK36* sequence that was not correlated with β-lactam resistance or porin loss. Therefore, reliance on WGS to determine porin functionality is not optimal. Another study of an *ompK36* amino acid variation demonstrated presence of the protein in the outer membrane with a constricted pore, resulting in reduced meropenem diffusion and resistance [44]. The role this modification played in resistance to other β-lactams is unknown. Decreased *phoE* expression in an imipenem- and meropenem-nonsusceptible *K pneumoniae* isolate with insertional inactivation of *ompK35* and *ompK36* has been noted [14]. The strains in the current study did not reveal any *PhoE* amino acid substitutions or significant changes in RNA expression.

The data presented from the panel of *K pneumoniae* clinical isolates and clones demonstrates that β-lactamase production, whether chromosomal or plasmid-encoded, and porin loss impact β-lactam resistance in an antibiotic-specific manner. Overall, this study shows that prediction of resistance based on phenotype among a class of antibiotics may not provide an accurate understanding of the mechanisms driving resistance in a given hospital. Several studies have repeatedly demonstrated genotype–phenotype mismatch in β-lactam resistance patterns, where isolates demonstrate β-lactam resistance beyond what would be expected for the given β-lactamase produced [34, 37, 48]. Carbapenem resistance, notably, occurs in isolates lacking carbapenemases, typically in the setting of an ESBL or pAmpC and porin loss [32, 49]. Furthermore, understanding the contribution of individual resistance mechanisms is difficult in clinical isolates given the genetic variability of the isolates [5]. Prior studies have employed isogenic clones and control for variability in the genetic background through several mutagenesis techniques [39, 48, 49] that lead to off-target effects. We have avoided some of these caveats by using the same vector and the natural promoter of the gene to express the β-lactamase.

The analyses carried out in the current study expand our understanding of pAmpC and ESBL-mediated resistance, without the complication of off-target consequences from nonspecific mutagenesis techniques. These data underscore the need to identify pAmpCs in clinical isolates. Finally, these data highlight the need for development of diagnostic tools capable of rapidly identifying the production of β-lactamases and porins in order to tailor antibiotic therapy as quickly as possible.

## Supplementary Data

Supplementary materials are available at *The Journal of Infectious Diseases* online (http://jid.oxfordjournals.org/). Supplementary materials consist of data provided by the author that are published to benefit the reader. The posted materials are not copyedited. The contents of all supplementary data are the sole responsibility of the authors. Questions or messages regarding errors should be addressed to the author.

## Supplementary Material

jiae204_Supplementary_Data

## Notes

***Financial support*.** This was an investigator-initiated study supported by Merck. Ceftolozane-tazobactam was provided by Merck. Funding to pay the Open Access publication charges for this article was provided by Creighton University School of Medicine.
