# Supplementary material for: What Contributes to the Minimum Inhibitory Concentration? Beyond β-Lactamase Gene Detection in Klebsiella pneumoniae
Source: J Infect Dis. 2024 Apr 24;230(4):e777–88. doi: 10.1093/infdis/jiae204 (PMC11481488; doi:10.1093/infdis/jiae204)
Supplement: jiae204_Supplementary_Data [file jiae204_supplementary_data.zip › Supplemental Data-Methods.docx]

**Supplemental Methods**

**Initial Screening of Clinical Isolates**

Our lab maintains a bio-repository of globally isolated gram-negative clinical isolates. Nineteen pAmpC and ESβL producing *K. pneumoniae* were selected for evaluation based on β-lactamase production and β-lactam MICs. Kp 23, a clinical isolate from 1973, prior to the introduction of third generation cephalosporins, was selected as the comparator due to its susceptible phenotype and lack of plasmid-encoded resistance genes. Clinical isolate KPM 20 was selected for cloning experiments due to its susceptible phenotype and undetectable OmpK35, OmpK36, and PhoE by western blot. The genomes for Kp 23 and KPM 20 have been published [1].

Isolates were initially screened for the β-lactamase genes CMY-2, ACC, EBC (including ACT and MIR), DHA, MOX, and FOX, CTX-M-15, CTX-M-14, NDM, KPC, IMP, VIM, and OXA-48 using the Streck ARM-D® *ampC* ID kit and ARM-D® b-lactamase ID kit. ESβLs in the TEM and SHV families were identified by isoelectric focusing (IEF) and sequencing. Additional β-lactamase genes not identified by PCR were identified by whole genome sequencing.

**Whole Genome Sequencing**

DNA was extracted using the MagAttract HMW DNA kit (Qiagen) or the DNeasy Blood and Tissue kit (Qiagen) gram-negative bacteria protocol. Isolates with a 260/230 ratio below 2 were purified by isopropanol precipitation. DNA was quantified by Qubit HS DNA assay. Sequencing libraries were prepared using the Illumina Nextera Flex DNA kit according to manufacturer instructions and verified by Bioanalyzer HS DNA assay (Agilent). Sequencing was performed using v2-300 cycle cartridges on the Illumina MiSeq. Trimmomatic PE [2] was used to trim adapter sequences and filter low quality reads. Gene sequences were collected using aTRAM [3]. β-lactamase genes were compared to RefSeq sequences (NCBI). TolC and porin mutations were compared to Kp 23 [1].

Not all the WGS data match exactly with the PCR data. PCR data cannot identify the specific allele of a β-lactamase such as CTX-M-28 for example. These differences were noted in the footnote of Table S4.

**RNA Isolation and qRT-PCR**

RNA isolation was performed using Trizol-Max (Invitrogen) as described previously [4]. RT-qPCR was performed with the QuantiNova SYBR Green RT-PCR kit (Qiagen) and amplified using a RotorGeneQ thermocycler. Fold change in RNA expression was calculated using the 2 ^-DDCt^ method, with *frr* as a housekeeping gene and Kp 23 as a comparator. Data represent n=3 with a ≤ 10% coefficient of variance. Primer sequences are available in Table S1. CTX-M-14 and CTX-M-15 primers published previously [5].

**Protein Isolation and Western Analysis**

Protein was isolated as described by Suelter, et. al [6]. Proteins were quantified by Pierce BCA (ThermoScientific). Outer membrane proteins were further purified by ultracentrifugation as described by Masuda, et. al [6, 7].

**Cloning of β-lactamase Genes**

CMY-2, CTX-M-14, and CTX-M-15 plasmids containing both the structural gene and promoter in pMDR009 (Table S3) [5, 8] were transformed into Kp 23 and KPM 20 by electroporation (Figure S2) [9]. Positive clones were confirmed by endpoint PCR and ESβL testing for CTX-M-14 and CTX-M-15. SHV-5 and DHA-AmpR clones were generated using pACYC-184 [10] (Figure S2, plasmid maps modified from [5, 8]) with the cloning primers in Table S1 and pCR-Blunt (Invitorgen) as a shuttle vector. Misc 289, an *E. coli* isolate transformed from the original SHV-5 clinical plasmid [11] and KPM 21, an isolate with inducible expression, were used as the source of SHV-5 and DHA-AmpR, respectively. Clones of DHA in the absence of *ampR* were lethal (data not shown). Kp 23 and KPM 20 were made competent by the Hanahan method [12] and transformed by heat shock. SHV-5 carriage was confirmed by isolation of the plasmid and ESβL confirmatory test. DHA-AmpR clones were confirmed by endpoint PCR.

**References Cited for Supplemental Data**

1. Maclean AKW, Hanson ND. Draft Genome Sequences of the Clinical Isolates Kp 23 and KPM 20. Microbiol Resour Announc **2021**; 10:e00119-21.

2. Anthony M. Bolger ML, Bjoern Usadel. Trimmomatic: a flexible trimmer for Illumina sequence data. Bioinformatics **2014**; 30:2114-20.

3. Allen JM, Huang DI, Cronk QC, Johnson KP. aTRAM - automated target restricted assembly method: a fast method for assembling loci across divergent taxa from next-generation sequencing data. BMC Bioinform **2015**; 16:98.

4. Schmidtke AJ, Hanson ND. Model system to evaluate the effect of ampD mutations on AmpC-mediated beta-lactam resistance. Antimicrob Agents Chemother **2006**; 50:2030-7.

5. Geyer CN, Fowler RC, Johnson JR, et al. Evaluation of CTX-M Steady-State mRNA, mRNA Half-Life and Protein Production in Various STs of *Escherichia coli*. J Antimicrob Chemother **2016**; 71:607-16.

6. Suelter CS, Hanson ND. OmpC Regulation Differs between ST131 and Non-ST131 *Escherichia coli* Clinical Isolates and Involves Differential Expression of the Small RNA MicC. J Antimicrob Chemother **2020**; 75:1151-8.

7. Masuda N, Sakagawa E, Ohya S. Outer Membrane Proteins Responsible for Multiple Drug Resistance in *Pseudomonas aeruginosa*. Antimicrob Agents Chemother **1995**; 39:645-9.

8. Reisbig MD. Mechanisms of Regulation and Resistance for Plasmid-Encoded *ampC* β-Lactamase Genes. Vol. PhD Dissertation. Graduate School of Creighton University, **2003**.

9. Michael R Green, Sambrook J. Transformation of *E. coli* by Electroporation. In: John Inglis AB, Alexander Gann, Judy Cuddihy, Kaaren Janssen, Michael Zierler, Kathleen Bubbeo, ed. Molecular Cloning, A Laboratory Manual. Vol. 1. Cold Spring Harbor, New York: Cold Spring Harbor Laboratory Press, **2012**.

10. Chang ACY, Cohen SN. Construction and Characterization of Amplifiable Multicopy DNA Cloning Vehicles Derived from the P15A Cryptic Miniplasmid. J Bacteriol **1978**; 134:1141-56.

11. L. Gutmann BF, F. W. Goldstein, N. Rizk, E. Pinto-Schuster, J. F. Acar, and E. Collatz. SHV-5, a Novel SHV-Type β-Lactamase That Hydrolyzes Broad-Spectrum Cephalosporins and Monobactams. Antimicrob Agents Chemother **1989**; 33:951-6.

12. Hanahan D. Studies on Transformation of *Escherichia coli* with Plasmids. J Mol Biol **1983**; 168:557-80.
