# Supplementary material for: What Contributes to the Minimum Inhibitory Concentration? Beyond β-Lactamase Gene Detection in Klebsiella pneumoniae
Source: J Infect Dis. 2024 Apr 24;230(4):e777–88. doi: 10.1093/infdis/jiae204 (PMC11481488; doi:10.1093/infdis/jiae204)
Supplement: jiae204_Supplementary_Data [file jiae204_supplementary_data.zip › Supplementary Figure 1 Legend.docx]

**Figure S1. OMP Representative Blots.** Blots were incubated in SuperSignal West Femto Maximum Sensitivity Substrate (ThermoScientific) for 2 minutes and then imaged using a 90 second exposure. (A) Anti-OmpK35 antibody was blotted against outer membrane fractions of clinical isolates of *K. pneumoniae.* (B) Anti-OmpK36 antibody was blotted against outer membrane fractions of clinical isolates of *K. pneumoniae.* (C) Anti-PhoE antibody was blotted against outer membrane fractions of clinical isolates of *K. pneumoniae.*
