# Supplementary material for: What Contributes to the Minimum Inhibitory Concentration? Beyond β-Lactamase Gene Detection in Klebsiella pneumoniae
Source: J Infect Dis. 2024 Apr 24;230(4):e777–88. doi: 10.1093/infdis/jiae204 (PMC11481488; doi:10.1093/infdis/jiae204)
Supplement: jiae204_Supplementary_Data [file jiae204_supplementary_data.zip › Supplementary Figure 2 Legend.docx]

**Figure S2**: **Cloning Plasmid Maps.** (top) pACYC-184 is a low copy vector used for cloning DHA-AmpR, SHV-5 and the empty vector control. (bottom) pMDR009 is a modified from pACYC-184 lacking the chloramphenicol promotor. It was used for cloning CMY-2, CTX-M-14, and CTX-M-15.
