# Supplementary material for: What Contributes to the Minimum Inhibitory Concentration? Beyond β-Lactamase Gene Detection in Klebsiella pneumoniae
Source: J Infect Dis. 2024 Apr 24;230(4):e777–88. doi: 10.1093/infdis/jiae204 (PMC11481488; doi:10.1093/infdis/jiae204)
Supplement: jiae204_Supplementary_Data [file jiae204_supplementary_data.zip › Supplementary Figure 3 Legend.docx]

**Figure S3: OXA-1 Protein Production.** OXA-1 protein determined by Western Blot in susceptible and non-susceptible clinical isolates in whole cell protein lysates.
