# Supplementary material for: What Contributes to the Minimum Inhibitory Concentration? Beyond β-Lactamase Gene Detection in Klebsiella pneumoniae
Source: J Infect Dis. 2024 Apr 24;230(4):e777–88. doi: 10.1093/infdis/jiae204 (PMC11481488; doi:10.1093/infdis/jiae204)
Supplement: jiae204_Supplementary_Data [file jiae204_supplementary_data.zip › Supplementary Table 1.docx]

**Table S1: Real Time and Cloning Primers**

| **Primer Target** | **Primer Sequence** |
| --- | --- |
| **Real Time** | |
| FRR Forward | GGACACGAATATCGCTGC |
| FRR Reverse | CGGGATTGTTGTTGAATACTACG |
| DHA Forward | ATGCCGTATGAGCAGTTGC |
| DHA Reverse | TCACGCCGTAAGATTCCG |
| CMY-2 Forward | CGTTAATCGCACCATCACC |
| CMY-2 Reverse | CGTCTTACTAACCGATCCTAGC |
| CTX-M-14 Forward | CGTTTCGTCTGGATCGCAC |
| CTX-M-14 Reverse | GCTGGGTAAAATAGGTCACC |
| CTX-M-15 Forward | CCGTCACGCTGTTGTTAGG |
| CTX-M-15 Reverse | GCTGTGTTAATCAATGCCACAC |
| SHV Forward* | ACAAGGTCACCCGCCTTGACC |
| SHV Reverse* | CGCTCAGACGCTGGCTGG |
| **Cloning** | |
| DHA-AmpR Forward | TGCCCGATACTCTCATCC |
| DHA-AmpR Reverse | GTGAAGGTGATGATTTGCGG |
| SHV-5 EcoR1 Forward | CCGGAATTCCGGAATTCGGTAGCTCAACGG |
| SHV-5 EcoR1 Reverse | CCGGAATTCCGGTTAGCGTTGCCAGTG |

*Due to sequence similarities between SHV alleles, primers detect all SHV subtypes
