# Supplementary material for: What Contributes to the Minimum Inhibitory Concentration? Beyond β-Lactamase Gene Detection in Klebsiella pneumoniae
Source: J Infect Dis. 2024 Apr 24;230(4):e777–88. doi: 10.1093/infdis/jiae204 (PMC11481488; doi:10.1093/infdis/jiae204)
Supplement: jiae204_Supplementary_Data [file jiae204_supplementary_data.zip › Supplementary Table 2.docx]

**Table S2. Antibody Sequences**

| **Antigen** | **Clonality** | **Antigen Type** | **Epitope** |
| --- | --- | --- | --- |
| **OmpK35** | Polyclonal | Full Protein | Full mature protein sequence |
| **OmpK36** | Polyclonal | Full Protein | Full mature protein sequence |
| **PhoE** | Polyclonal | Full Protein | Full mature protein sequence |

Antibodies were generated by GenScript in New Zealand Rabbits. Antibodies were isolated by protein A selection and verified by ELISA at GenScript. Antibodies were further validated by blotting against purified protein, knockout strains of the specific porin, and for reactivity against the other two porin proteins. The limit of detection is 0.5 µg for anti-PhoE, 1 µg for anti-OmpK35, and 5 µg for anti-OmpK36. Primary antibodies were diluted 1:30,000 and secondary antibodies 1:100,000.
