# Supplementary material for: What Contributes to the Minimum Inhibitory Concentration? Beyond β-Lactamase Gene Detection in Klebsiella pneumoniae
Source: J Infect Dis. 2024 Apr 24;230(4):e777–88. doi: 10.1093/infdis/jiae204 (PMC11481488; doi:10.1093/infdis/jiae204)
Supplement: jiae204_Supplementary_Data [file jiae204_supplementary_data.zip › Supplementary Table 3.docx]

**Table S3**: Cloning Strains

| **Strain** | **Cloning Vector** | **Copy Number*** | **Background** | **Porin Phenotype** | **β-Lactamase** |
| --- | --- | --- | --- | --- | --- |
| Kp 23 – EVC | pACYC-184 | Low | Kp 23 | Present | None |
| Kp 23 – SHV-5 | pACYC-184 | Low | Kp 23 | Present | SHV-5 |
| Kp 23 – DHA-AmpR | pACYC-184 | Low | Kp 23 | Present | DHA-AmpR |
| Kp 23 – CMY-2 | pMDR009 | Low | Kp 23 | Present | CMY-2 |
| Kp 23 – CTX-M-14 | pMDR009 | Low | Kp 23 | Present | CTX-M-14 |
| Kp 23 – CTX-M-15 | pMDR009 | Low | Kp 23 | Present | CTX-M-15 |
| KPM 20 – EVC | pACYC-184 | Low | KPM 20 | Absent | None |
| KPM 20 – SHV-5 | pACYC-184 | Low | KPM 20 | Absent | SHV-5 |
| KPM 20 – DHA-AmpR | pACYC-184 | Low | KPM 20 | Absent | DHA-AmpR |
| KPM 20 – CMY-2 | pMDR009 | Low | KPM 20 | Absent | CMY-2 |
| KPM 20 – CTX-M-14 | pMDR009 | Low | KPM 20 | Absent | CTX-M-14 |
| KPM 20 – CTX-M-15 | pMDR009 | Low | KPM 20 | Absent | CTX-M-15 |

*pACYC-184 and its derivative, pMDR009, have a p15 ORI with copy numbers between 15 and 20 copies.
