# Supplementary material for: What Contributes to the Minimum Inhibitory Concentration? Beyond β-Lactamase Gene Detection in Klebsiella pneumoniae
Source: J Infect Dis. 2024 Apr 24;230(4):e777–88. doi: 10.1093/infdis/jiae204 (PMC11481488; doi:10.1093/infdis/jiae204)
Supplement: jiae204_Supplementary_Data [file jiae204_supplementary_data.zip › Supplementary Table 4.docx]

**Table S4**: PCR identified beta-lactamases vs. Whole genome sequencing beta-lactamases

| **Isolate** | **β-Lactamases by PCR** | **β-Lactamases by WGS** |
| --- | --- | --- |
| Kp 23 | None | SHV-11 |
| KPM 1 | None | None |
| KPM 5 | CMY-2-like, CTX-M-14-like | CTX-M-14, SHV-11, TEM-1 |
| KPM 8 | CTX-M-15 | CTX-M-15, SHV-27, TEM-1 |
| KPM 9 | CTX-M-15 | CTX-M-15, SHV-27, TEM-1 |
| KPM 10 | CTX-M-15-like | CTX-M-15, OXA-1, SHV-28, TEM-1 |
| KPM 17 | CTX-M-15-like | CTX-M-15, OXA-1, SHV-11, TEM-1 |
| KPM 18 | CTX-M-15-like | CTX-M-15, SHV-28, OXA-1, TEM-1 |
| KPM 20 | SHV-1** | SHV-11 |
| KPM 21 | DHA | DHA*, SHV-60 |
| KPM 23 | CMY-2 | CMY-2, SHV-187, TEM-1 |
| KPM 26 | CTX-M-19 | CTX-M-19, SHV-1, TEM-1 |
| KPM 29 | CMY-2 | CMY-31, SHV-5 |
| KPM 30 | CTX-M-28*** | CTX-M-15, OXA-1, TEM-1, SHV-1 |
| KPM 32 | TEM-1, SHV-1, CTX-M-15 | CTX-M-15, OXA-1, SHV-12 |
| KPM 42 | DHA | DHA-1, SHV-12, TEM-1 |
| KPM 43 | CTX-M-14, DHA-1 | CTX-M-14, DHA-1, SHV-11, TEM-1 |
| KPM 44 | CTX-M-14, DHA-1 | CTX-M-14, DHA-1, SHV-11, TEM-1 |
| KPM 60 | CTX-M-15, TEM-1, SHV-1 | CTX-M-15, OXA-1, SHV-11, TEM-1 |
| KPM 61 | CTX-M-15 | CTX-M-15, OXA-1, SHV-1, TEM-1 |

*DHA sequence does not fully align with any known allele

**SHV and TEM identified by iso-electric focusing

**Identified by Sanger Sequencing
